# Atezolizumab and Bevacizumab Induced Diabetes Mellitus and Myocarditis: A Case Report

**DOI:** 10.7759/cureus.97311

**Published:** 2025-11-20

**Authors:** Muhammad A Mirza, Vimalraj Periyasami, Amail Kasi, Keerthana Pampapathi, Satyanarayana V Sagi

**Affiliations:** 1 Internal Medicine, Peterborough City Hospital, Peterborough, GBR; 2 Acute Medicine, Peterborough City Hospital, Peterborough, GBR; 3 Medicine, Peterborough City Hospital, Peterborough, GBR; 4 Endocrinology and Diabetes, Peterborough City Hospital, Peterborough, GBR

**Keywords:** atezolizumab plus bevacizumab therapy, drug-induced myocarditis, immune check-point inhibitor, multidisciplinary intervention, type i diabetes mellitus

## Abstract

Checkpoint inhibitors such as atezolizumab and bevacizumab have improved outcomes in hepatocellular carcinoma (HCC), but their use can lead to severe immune-related adverse events (IRAEs). We report the case of a 71-year-old man with HCC and multiple comorbidities who developed diabetic ketoacidosis (DKA) secondary to new-onset type 1 diabetes mellitus and myocarditis, five days after his second cycle of atezolizumab and bevacizumab. Laboratory findings demonstrated metabolic acidosis, hyperglycemia, ketonemia, elevated troponin, B-type natriuretic peptide (BNP), C-reactive protein (CRP), deranged liver function, and acute kidney injury. ECG revealed a new right bundle branch block, and echocardiography confirmed impaired left ventricular function. He was managed with standard DKA guidelines, transitioned to a basal-bolus insulin regimen, and commenced on high-dose corticosteroids for myocarditis, with multidisciplinary input from oncology and cardiology teams. This case highlights the importance of early recognition and coordinated management of IRAEs, as well as the need for long-term surveillance for endocrine and cardiac complications following checkpoint inhibitor therapy.

## Introduction

Hepatocellular carcinoma (HCC) is a leading cause of cancer-related mortality worldwide, with limited therapeutic options for advanced stages. The advent of immune checkpoint inhibitors (ICIs) and anti-angiogenic agents, such as atezolizumab (anti-programmed death-ligand 1 (PD-L1)) and bevacizumab (anti-vascular endothelial growth factor (VEGF)), has revolutionized first-line treatment for unresectable HCC. The landmark IMbrave150 trial demonstrated superior survival benefits with this combination over sorafenib, establishing it as the new standard of care [1]. However, these biologics are associated with immune-related adverse events (irAEs), which can affect multiple organ systems, including endocrine and cardiovascular systems [2].

While the efficacy of atezolizumab-bevacizumab in HCC is well-documented, particularly its favourable organ-specific response rates in both intrahepatic and extrahepatic lesions [3], the risk of severe irAEs remains a critical concern. For instance, immune-checkpoint inhibitor (ICI)-induced type 1 diabetes mellitus (T1DM) and myocarditis are rare but increasingly recognized complications, as highlighted in recent case reports [4]. These irAEs often present acutely, with T1DM manifesting as diabetic ketoacidosis (DKA) and myocarditis as cardiac dysfunction or arrhythmias, necessitating immediate intervention.

This case report describes a 71-year-old male patient with HCC who developed T1DM with DKA and myocarditis within days of his second atezolizumab-bevacizumab cycle, highlighting the aggressive timeline of irAEs. His presentation underscores the diagnostic challenges in distinguishing irAEs from sepsis or metabolic derangements, as well as the imperative for early multidisciplinary intervention. High-dose corticosteroids, insulin therapy, and heart failure management stabilized the patient, but long-term monitoring is essential given the potential for persistent cardiomyopathy and diabetes-related complications.

By reporting this case, we aim to emphasize the clinical significance of promptly identifying the risks of developing the rapid onset of ICI-induced T1DM and myocarditis, along with prompt treatment in patients receiving combination immunotherapy, and to contribute to the growing literature on irAEs, which remains sparse for this specific dual complication in HCC patients.

## Case presentation

A 71-year-old male patient presented to the emergency department with confusion, fluctuating consciousness, and agitation over the past two days. Confusion was first noticed by family members, initially manifesting as aggression, which progressively worsened to impaired orientation and communication difficulties. His symptoms began five days after receiving his second cycle of immunotherapy, consisting of atezolizumab and bevacizumab sessions every third week, for advanced hepatocellular carcinoma. He denied any recent illnesses such as diarrhea, vomiting, cough, breathlessness, or urinary symptoms. In the past, he had been treated for right tonsillar squamous cell carcinoma (SCC) Stage T2 N1 M0, P16 positive, which had previously been treated with external beam radiotherapy completed two years ago, and was in remission. Other medical history included liver cirrhosis, oesophageal varices, deep vein thrombosis, hypertension, and hypothyroidism. His regular medications included bendroflumethiazide, finasteride, thyroxine, tamsulosin, and nystatin. He lived alone and independently with regular activities of daily living.

On admission, his clinical examination on admission revealed confusion and agitation, with a Glasgow Coma Scale (GCS) score of 11/15. A full neurological examination was unremarkable. His cardiovascular, respiratory, and abdominal examinations were unremarkable except for marked pitting edema up to the shins bilaterally. His initial vital signs showed hypotension (96/59 mmHg) with tachycardia (102 beats per minute). Respiratory rate was noted to be 22 per minute, with oxygen saturations being 95% at room air. He was afebrile.

Laboratory investigations showed a raised capillary glucose of 36.0 mmol/L and capillary ketones of 3 mmol/L, and venous blood gas (VBG) revealed a pH of 7.28, bicarbonate of 16.9, and lactate of 14.9. Interestingly, his HbA1c, measured 15 days prior, was 31 mmol/mol and within normal limits. Electrolytes showed sodium of 129 mmol/L and potassium of 5.5 mmol/L. His renal function indicated acute kidney injury, with a raised creatinine of 149 µmol/L and an estimated glomerular filtration rate (eGFR) of 40 ml/minute/1.73m². Urea was 15.8 mmol/L. Previous renal function, including urea, had been normal. Investigation results are listed in Table 1.

**Table 1:** Laboratory investigations eGFR: estimated glomerular filtration rate; HbA1c: glycated hemoglobin

Investigation	Patient value	Reference range
Capillary glucose	36 mmol/L	4.0–7.8 mmol/L
Capillary ketone	3 mmol/L	<0.6 mmol/L
pH	7.28	7.35–7.45
Bicarbonate	16.9 mEq/L	22–28 mEq/L
Lactate	14.9 mmol/L	0.5–2.2 mmol/L
HbA1c	31 mmol/mol	20–41 mmol/mol
Sodium	129 mmol/L	135–145 mmol/L
Potassium	5.5 mmol/L	3.5–5.0 mmol/L
Creatinine	149 µmol/L	60–110 µmol/L
eGFR	40 ml/minute/1.73 m²	>90 mL/minute/1.73 m²
Urea	15.8 mmol/L	2.5–7.8 mmol/L
1st Troponin T	241 ng/L	<14 ng/L
2nd Troponin T	361 ng/L	<14 ng/L
Prothrombin time	18.5 seconds	11–15 seconds
International normalized ratio	1.55	0.8–1.2
Fibrinogen	1.6 g/L	2.0–4.0 g/L
C-reactive protein	38 mg/L	<5 mg/L
Lymphocytes	0.6×10⁹ cells per litre	1.0–3.5 ×10⁹ cells per litre

Islet cell antibody (ICA) and islet antigen-2 (IA-2) antibody tests were negative. His anti-glutamic acid decarboxylase (GAD) antibody level was 37.7 IU/mL (reference value, <10), indicating underlying autoimmunity. Serial troponin T levels were elevated, rising from 241 ng/L to 361 ng/L. Initial blood tests revealed a deranged coagulation profile, with prothrombin time (PT) of 18.5 seconds, international normalized ratio (INR) of 1.55, and fibrinogen of 1.6 g/L. C-reactive protein (CRP) was 38 mg/L, platelets were low, and liver function test was abnormal, consistent with his chronic liver disease at his baseline. Lymphocytes were noted to be 0.6 ×10⁹ cells per litre.

**Figure 1:**
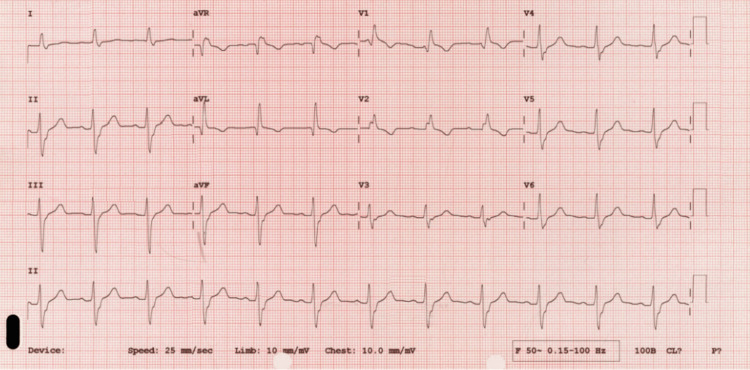
Electrocardiogram showing sinus rhythm with a new right bundle branch block (RBBB)

**Figure 2:**
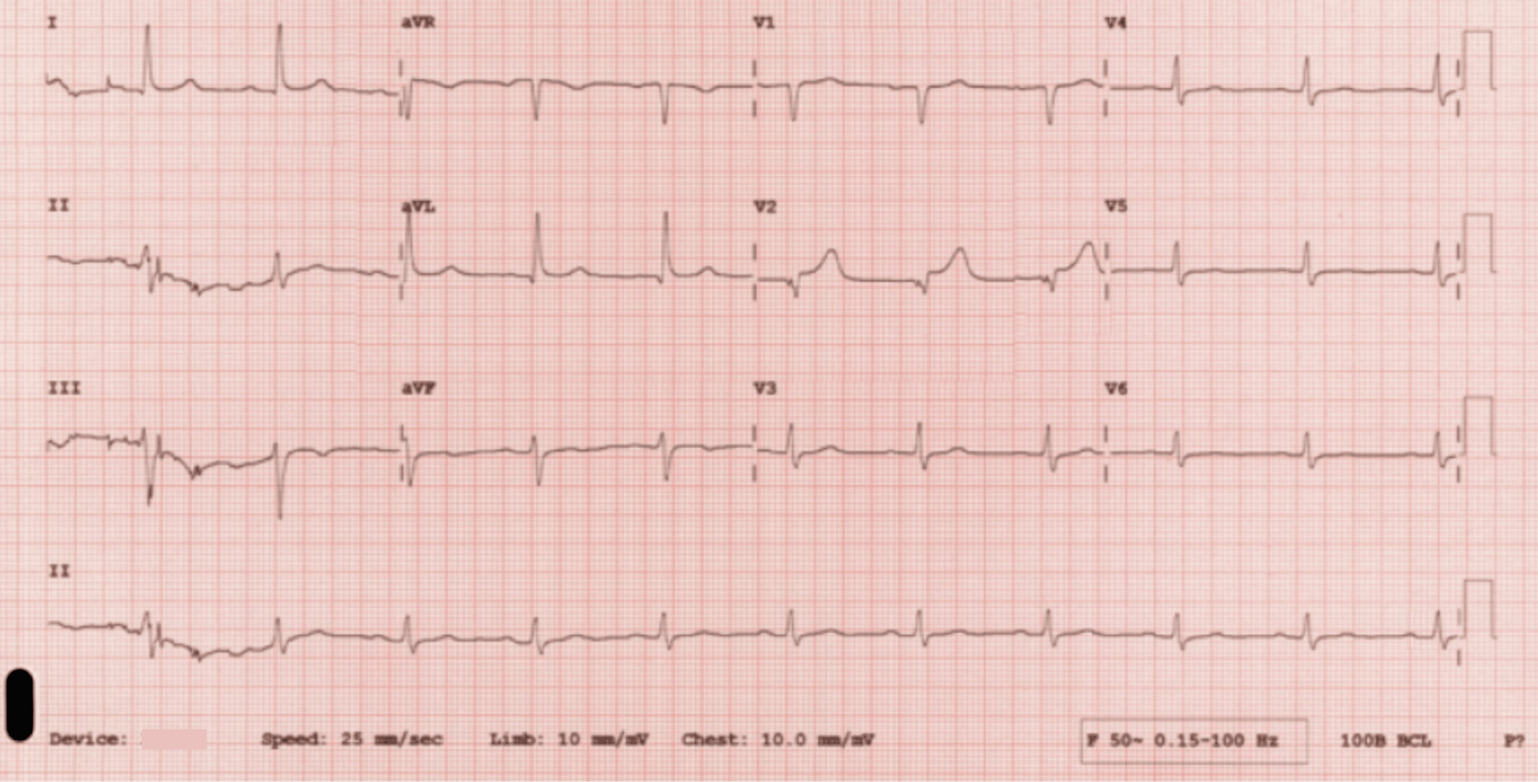
Subsequent electrocardiogram which returned normal

Electrocardiogram (ECG) (Figure 1) showed sinus rhythm with a new right bundle branch block (RBBB), subsequently returned to normal (Figure 2). A computed tomography (CT) of the head showed no radiological features of acute intracranial pathology.

Based on investigations, the diagnoses of DKA and decompensated heart failure, all suspected to be secondary to irAEs. Positive antibodies supported immune-mediated beta-cell destruction as the mechanism for immunotherapy-induced T1DM, due to the absence of classic autoimmune markers like ICA and IA2. While the patient’s anti-GAD antibody titres were mildly elevated (37 IU/mL; normal <10), this likely reflects ICI-mediated disruption of immune tolerance, rather than pre-existing autoimmune diabetes.

The abrupt onset of DKA within days of ICI initiation, coupled with normal HbA1c prior to therapy, strongly supports a drug-induced immune mechanism. Anti-GAD antibodies may arise transiently in ICI-induced diabetes due to epitope spreading or bystander activation of autoreactive T cells, distinct from traditional Type 1 diabetes pathogenesis [5]. With regard to ECG changes and raised troponins, a presumptive diagnosis of myocarditis likely induced by recent immunotherapy was made.

**Figure 3:**
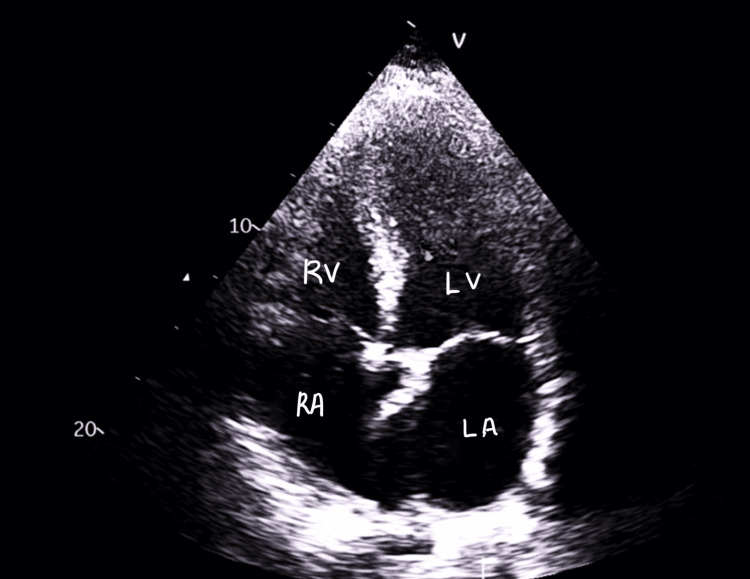
A four-chamber view shows markedly dilated atrias and ventricles RA: right atrium; RV: right ventricle; LA: left atrium; LV: left ventricle

Echocardiography (Figure 3) showed a dilated left ventricle (LV) by volume index, impaired LV systolic function with biplane ejection fraction (EF) of 41%, and peak average global longitudinal strain (GLS) of -14.2%. Global hypokinesis and impaired diastolic function with normal filling pressures were noted, along with a dilated left atrium (LA), and mild to moderate mitral regurgitation (MR) and mild tricuspid regurgitation (TR).

On Day 1 of admission, the oncology team reviewed the patient and attributed the events to immunotherapy toxicity. As per Clatterbridge immunotherapy toxicity guidelines [6], the patient was initially started on methylprednisolone 1 mg/kg/day with a proton pump inhibitor (PPI). On Day 3, the patient showed gradual clinical improvement, evidenced by improved alertness and orientation compared to the time of admission. Considering a reassuring response, methylprednisolone was increased to 2 mg/kg/day. DKA was managed according to the hospital’s DKA guidelines.

On Day 2, the endocrinology team reviewed the patient and confirmed a diagnosis of DKA secondary to T1DM induced by immunotherapy, a permanent condition. The patient’s metabolic derangement improved with DKA management, and he was later initiated on a basal-bolus insulin regimen of glargine (Toujeo) and aspart (Novorapid). On Day 3, after the echo was done, the cardiology team reviewed the patient and concurred with the diagnosis of immunotherapy-induced myocarditis. Management included IV diuretics, later switched to oral diuretics upon discharge. The patient was also started on an angiotensin converting enzyme (ACE) inhibitor, beta-blocker, and mineralocorticoid receptor antagonist (MRA) spironolactone, to be up-titrated as tolerated.

Management with IV methylprednisolone, along with treatments for T1DM and myocarditis, resulted in marked clinical improvement and resolution of all presenting symptoms. On Day 7, fluctuating consciousness resolved, and the patient was fully alert and oriented, at which point IV methylprednisolone was switched to oral prednisolone 75 mg. Oral prednisolone 75 mg was planned to be gradually weaned off (5 mg each week) to a maintenance dose of 40 mg until reviewed by the oncology outpatient department in four weeks. Immunotherapy-induced T1DM was well controlled with the basal-bolus insulin regimen. The patient was reviewed by a dietitian for advice on managing new-onset diabetes and planned for follow-up with diabetic specialist nurses to optimize diabetic control.

The myocarditis responded well, with improvement in peripheral edema using IV diuretics, later switched to oral diuretics with continuation of ACE inhibitors, beta-blockers, and spironolactone. A community heart failure nurse follow-up was arranged to optimize heart failure medications. A repeat echocardiogram was booked in six weeks to review whether cardiac changes are permanent. Full blood count (FBC) at discharge showed improved platelets at 76 × 10⁹/L. Liver function tests, renal function, and electrolytes had returned to baseline at discharge. On Day 10, the patient achieved 80% of his baseline prior ongoing illness and was medically optimised for discharge with further follow-ups as mentioned above.

**Figure 4:**
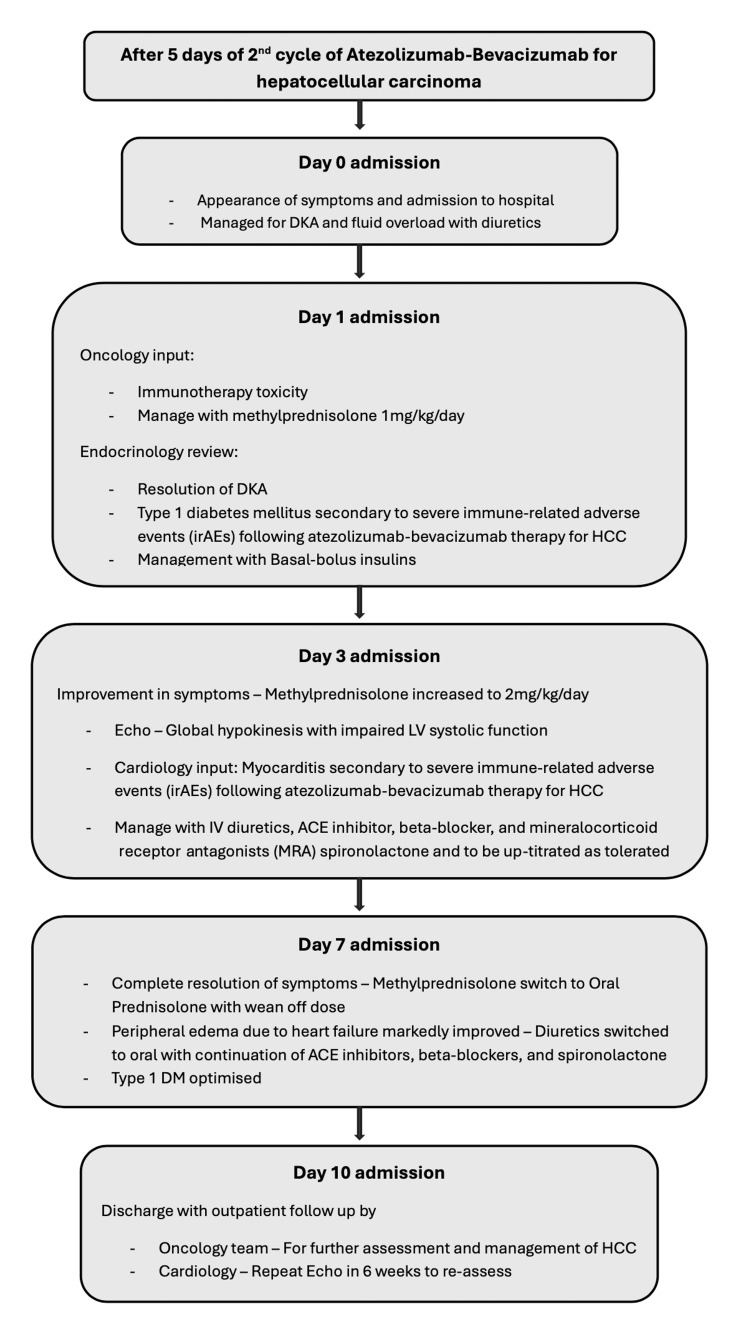
Summary of clinical course during admission DKA: diabetic ketoacidosis; HCC: hepatocellular carcinoma; ACE: angiotensin converting enzyme; DM: diabetes mellitus

Figure 4 illustrates the sequence of events during the admission, outlining the case presentation and progression

## Discussion

ICIs are an important advancement in cancer treatment, with manageable toxicity profiles and improved survival outcomes in a series of advanced malignancies, such as melanoma, non-small cell lung cancer, HCC, renal cell carcinoma, and Hodgkin lymphoma [7]. HCC is a common cancer globally with fatal outcomes. While early stages can be cured by resection, transplantation, or ablation, many patients present with unresectable disease. Combination therapy with atezolizumab and bevacizumab is used in patients with unresectable HCC [8]. According to a phase 3 randomized trial (IMBrave150) in HCC patients with Child-Pugh class A cirrhosis, the hazard ratio for overall survival was 0.58 in favor of atezolizumab and bevacizumab; i.e., there was a 42% reduction in the risk of death with the combination compared with sorafenib [9].

ICIs work principally by overcoming immune tolerance against the tumor, mediated by inhibitory molecules such as PD-1, PD-L1, or cytotoxic T-lymphocyte-associated protein 4 (CTLA-4), and enhancing the body's own immune response [10]. Atezolizumab is a humanized monoclonal antibody that works by inhibiting the interaction between PD-L1 (on tumor cells) and PD-1 (on T cells), leading to upregulation of the immune response against tumor cells. Bevacizumab, on the other hand, is an anti-VEGF antibody that also has an immunomodulatory effect on the immune microenvironment [8]. The occurrence of irAEs associated with ICIs presents a significant challenge in contemporary clinical practice.

A review of the literature revealed various irAEs associated with ICI use, including cardiac events (myocarditis, pericarditis, Takotsubo cardiomyopathy, acute coronary syndrome, and arrhythmias) [7], endocrine disorders (hypophysitis, thyroid dysfunction, and primary adrenal insufficiency) [11], encephalitis [10], acute interstitial nephritis [12], and corneal perforation [13]. Management of irAEs typically involves discontinuation of the ICI agent and initiation of immunosuppressive therapy with corticosteroids or other agents such as intravenous immunoglobulin (IVIG) or plasmapheresis.

T1DM is an uncommon but potentially life-threatening adverse event associated with ICI treatment, particularly when diagnosis and management are delayed. Although precise pathophysiological mechanisms remain unclear, the proposed hypothesis is that PD-1-PD-L1 pathway blockade leads to activation of autoreactive T cells that target pancreatic islet beta cells. Resulting immune-mediated destruction of these cells ensues with manifestations of diabetes [14,15]. Additionally, genetic predisposition plays a crucial role, with Major Histocompatibility Complex (MHC) and non-MHC genes influencing individual susceptibility. Various human leukocyte antigen (HLA) genotypes pose a high risk, such as HLA DR3-DQ2 or DR4-DQ8 double serotypes, found in 90% of patients with T1DM [16].

Myocarditis is a rare complication of ICI therapy, having poor outcomes, with 50% of cases resulting in fatality [17]. T cell infiltration is the main pathophysiological driver, hence making endomyocardial biopsy the gold standard for diagnosis. Fulminant cases have an early onset, presenting as arrhythmias and conduction disturbances [18]. Atezolizumab combined with bevacizumab has been available for the treatment of HCC since 2020 [19]. As a relatively recent therapeutic option, its use has been associated with adverse effects, though current literature on these irAEs remains limited. To date, only a small number of cases have been reported involving myocarditis and T1DM presenting as DKA. With the expanding indications for these agents, a rise in irAE-related presentations is anticipated in clinical settings. This underscores the need for clinical vigilance, early identification, and prompt management of such rare but potentially serious complications.

## Conclusions

This case highlights the rapid onset of severe irAEs such as T1DM with DKA and myocarditis, following atezolizumab-bevacizumab therapy for HCC. Early recognition and multidisciplinary management with corticosteroids, insulin, and cardiac support were critical to recovery. As ICI use expands, clinicians must be aware of the rare but life-threatening irAEs. This case underscores the need for patient education, close monitoring, and prompt intervention to balance therapeutic efficacy with toxicity risks. Long-term follow-up is essential to monitor for delayed autoimmune sequelae and optimize chronic disease management. Collaboration between oncologists, endocrinologists, and cardiologists plays a pivotal role in ensuring comprehensive care.
